# Identification of the *TLP* gene family in Rosaceae and function of PbrTLP28 in leaf disease resistance

**DOI:** 10.3389/fpls.2026.1990307

**Published:** 2026-10-28

**Authors:** Hao Zhang, Xueying Liu, Ruobing Sun, Huishu Yan, Chi Lan, Ziwen Xiao, Na Ma, Liqing Lu, Haiqi Zhang, Chunyan Liu, Peng Wang, Shaoling Zhang, Juyou Wu, Yongjie Qi, Zhenghui Gao

**Affiliations:** 1 Key Laboratory of Horticultural Crop Germplasm Innovation and Utilization (Co-construction by Ministry and Province), Anhui Provincial Key Laboratory for Germplasm Resources Creation and High-Efficiency Cultivation of Horticultural Crops, Institute of Horticulture, Anhui Academy of Agricultural Sciences, Hefei, Anhui, China; 2 Sanya Institute of Nanjing Agricultural University, National Key Laboratory of Crop Genetics & Germplasm Enhancement and Utilization, College of Horticulture, Nanjing Agricultural University, Nanjing, Jiangsu, China

**Keywords:** black spot disease, expression pattern, genome-wide identification, pear, TLP

## Abstract

Thaumatin-like proteins (TLPs) are induced by various biotic and abiotic signals, exhibit antifungal activity, and serve essential functions in plant development and stress defense. However, studies on the identification of *TLPs* in Rosaceae fruit trees and their specific functions in leaf disease resistance remain limited. In this study, 177 *TLPs* were characterized from five Rosaceae genomes (*Fragaria vesca*, *Malus domestica*, *Pyrus bretschneideri*, *Prunus mume*, and *Prunus persica*) and classified into 11 subfamilies (Groups 1-11) according to their phylogenetic and structural features. Diverse gene duplication modes facilitated the expansion of the *TLP* family, whereas purifying selection served as the primary driving force of *TLP* evolution. Notably, expression analysis revealed that *PbrTLP28* was significantly upregulated following black spot disease infection. Signal peptide prediction, conserved domain analysis, and subcellular localization assays confirmed that PbrTLP28 is a secreted protein. Functional assays demonstrated that silencing *PbrTLP28* attenuated the resistance of pear leaves to black spot disease, indicating that PbrTLP28 functions in disease resistance. Collectively, these findings provide significant insights into *TLP* gene function, expression, and evolution in Rosaceae.

## 1 Introduction

Thaumatin-like proteins (TLPs) are so named because they are highly similar to thaumatin. Thaumatin is a sweet protein extracted from the fruit of *Thaumatococcus daniellii* Benth (van der Wel and Loeve, 1972). Despite their high sequence homology, these two proteins exhibit distinct functional profiles: thaumatin is intensely sweet yet devoid of antifungal activity, whereas TLPs possess antifungal properties but lack sweetness (van Loon et al., 2006). TLPs are grouped into the pathogenesis-related protein family 5 (PR-5), which comprises TLPs, osmotins, and zeamatins (Zhao and Su, 2010; de Jesus-Pires et al., 2020).

The *TLPs* belong to a multigene family and contain a highly conserved thaumatin domain (Shatters et al., 2006; Zhang et al., 2024b). Most *TLPs* possess a conserved motif G-X-[GF]-X-C-X-T-[GA]-D-C-X(1,2)-[GQ]-X(2,3)-C (Liu et al., 2020), and are grouped by molecular weight into long-chain TLPs (L-TLPs) and short-chain TLPs (S-TLPs) (Liu et al., 2010). These two groups differ not only in amino acid number, molecular weight, and structure, but also in their natural distribution. L-TLPs are ubiquitously present in most organisms, including animals, plants, and insects (Sharma et al., 2020). They possess a molecular weight of 21–26 kDa and feature 16 conserved cysteine residues (Shatters et al., 2006). In contrast, S-TLPs have been identified exclusively in gymnosperms and monocotyledonous plants, with a smaller size (16–17 kDa) and only 10 conserved cysteines. These cysteines are critical for the formation of disulfide bonds, which not only stabilize the protein structure but also confer tolerance to high temperatures, pH extremes, and enzymatic degradation (Liu et al., 2010; Li et al., 2022). Furthermore, most TLPs feature an N-terminal signal sequence that targets them to the secretory system (Wang et al., 2010; Zhang et al., 2018a; Muoki et al., 2021). Some TLPs contain a C-terminal peptide that is thought to target the protein to the vacuole (Liu et al., 1996). To date, three-dimensional structure predictions of several plant TLPs have revealed that they generally comprise three functional domains (I, II, and III). Functional domain I forms the core of the entire molecule, with domains II and III flanking either side of domain I. A highly conserved V-shaped cleft (V-cleft) between domains I and II is critical for the catalytic activity of these proteins (Zhang et al., 2024b). Based on their isoelectric points, TLPs are categorized as acidic, neutral, or basic. Current research suggests that the V-clefts of antifungal TLPs are predominantly acidic, which is attributed to the presence of five conserved REDDD residues within this region (Liu et al., 2010; Marangon et al., 2014). Moreover, the highly acidic V-cleft of TLPs is closely associated with their antifungal and β-glucanase activities (Zhang et al., 2018b). Collectively, these conserved structural features directly govern the various biological roles of TLPs.

TLPs exhibit diverse biological activities, such as antifungal, β-1,3-glucanase, allergenic, and enzyme-inhibitory (Zhang et al., 2024b). Among these, antifungal activity represents the primary biological function. In plants, TLPs primarily inhibit fungal infection by lysing fungal spores, suppressing spore germination, and reducing the viability of nascent hyphae (Jiao et al., 2018). *In vitro* antifungal assays have demonstrated that the AdTLP protein from peanut inhibits spore germination and hyphal growth in multiple fungal species (Singh et al., 2013). ObTLP1 protein from *Ocimum basilicum* suppresses mycelial growth in plant-pathogenic fungi (Misra et al., 2016). VVTL-1, a protein derived from grapevine, significantly inhibits germination of spores and hyphal elongation of *Erysiphe ampelina* (Jayasankar et al., 2003). ClTLP27, a watermelon‐derived protein, potently suppresses the hyphal growth of multiple fungi, including *Fusarium oxysporum* (Zhang et al., 2018b). *In vivo*, overexpression of *VqTLP29* in Arabidopsis conferred stronger resistance against powdery mildew and *Pseudomonas syringae* (Yan et al., 2017). Overexpression of *AsPR5* significantly improved tolerance to *B. cinerea* in both garlic and Arabidopsis (Rout et al., 2016). Expression of the *OsTLP* gene in cassava delayed the onset of anthracnose and enhanced disease resistance (Ojola et al., 2018), while *PeTLP* overexpression in poplars improved resistance to spot disease (Sun et al., 2020). Concurrently, pathogen challenge induces *TLP* gene expression. For instance, treatment of *Gossypium barbadense* with *Verticillium dahliae* significantly upregulated *GbTLP* expression (Zhang et al., 2021), and inoculation of melon with powdery mildew also significantly upregulated *CmTLPs* (Liu et al., 2020). Furthermore, TLPs exert antifungal effects via their β‐1,3‐glucanase activity. They bind β‐1,3‐glucan, a major fungal wall component, and degrade it, thereby disrupting cell wall integrity or altering membrane permeability to exert fungicidal effects (Grenier et al., 1999; Liu et al., 2010). However, the antifungal activity of TLPs is not always positively linked to their β‐1,3‐glucanase activity. For example, TLPs from cherry and apple exhibit moderate β-1,3-glucanase activity yet no antifungal action (Menu-Bouaouiche et al., 2003), indicating that this enzymatic property is not the exclusive determinant of antifungal effects.

TLPs not only exhibit diverse enzymatic activities but also induce programmed cell death in phytopathogens. For example, the TLP from banana inhibits the growth of *Penicillium expansum* by inducing pathogen cell rupture and elevating membrane permeability, which eventually results in cell death (Jiao et al., 2018). In addition, studies have demonstrated that synthetic TLP peptides can inhibit the growth of *Candida albicans* and *Saccharomyces cerevisiae*. Further investigations revealed that TLP peptides bind to specific receptors on the cell membranes of *C. albicans* and *S. cerevisiae*, thereby triggering a reactive oxygen species burst and subsequent apoptosis (Lopes et al., 2019). Moreover, TLPs enhance plant responses to abiotic stresses. For example, heterologous *ObTLP1* expression in Arabidopsis enhances salt and drought tolerance (Misra et al., 2016). In winter rye leaves, TLPs enhance plant cold resistance (Yu and Griffith, 1999). Thus, TLPs can enhance plant tolerance to abiotic stresses and participate in processes such as pollination (O'Leary et al., 2007), flowering time regulation (Munis et al., 2010), and seed germination (Seo et al., 2008).

Genome sequencing of multiple Rosaceae fruit trees has established a solid groundwork for intensive study of the *TLP* family within Rosaceae. Here, we systematically analyzed *TLPs* in pear and four other Rosaceae genomes. We also found that PbrTLP28, a secreted protein abundantly expressed in leaves, critically contributes to pear resistance. Our work provides valuable insights and candidate resistance genes for future functional research on TLPs in Rosaceae fruit trees.

## 2 Materials and methods

### 2.1 Characterization and physicochemical analysis of TLP protein

Two strategies were employed to characterize *TLP* family members across five Rosaceae genomes. First, we performed hidden Markov model searches (E-value < 1e^-5^) against each genome using the thaumatin (PF00314) domain. Then, we carried out BLASTP searches with Arabidopsis TLP proteins as queries. All putative *TLPs* were validated for the thaumatin domain using SMART (https://smart.embl.de/) and InterPro (http://www.ebi.ac.uk/interpro/). In total, 177 *TLPs* were identified from the five Rosaceae genomes. Genome data for apple (*Malus domestica*, v1.1), peach (*Prunus persica*, v2.1), pear (*Pyrus bretschneideri*, v1.0), plum (*Prunus mume*, v1.0), and strawberry (*Fragaria vesca*, v4.0) were retrieved from the GDR platform (https://www.rosaceae.org/) (Velasco et al., 2010; Zhang et al., 2012; Verde et al., 2013; Wu et al., 2013; Li et al., 2019). We further estimated the physicochemical characteristics of all detected TLP proteins via the ExPASy-ProtParam (http://web.expasy.org/protparam/).

### 2.2 Bioinformatics analysis of the *TLPs* in Rosaceae

To analyze the phylogenetic relationships of TLP proteins across Rosaceae, rice, and Arabidopsis, we inferred a phylogenetic tree with the neighbor-joining method in MEGA 7.0, supported by 1,000 bootstrap replicates (Kumar et al., 2016). Gene structures and conserved motifs of TLPs were separately analyzed using GSDS 2.0 (https://gsds.gao-lab.org/) and MEME v5.5.8 (https://meme-suite.org/meme/tools/meme). Signal peptides and subcellular localizations were predicted via SignalP-5.0 (https://services.healthtech.dtu.dk/services/SignalP-5.0/) and CELLO v.2.5 (https://cello.life.nctu.edu.tw/). Chromosomal locations of *TLPs* in Rosaceae were extracted from genome annotation data. Synteny analysis of Rosaceae *TLPs* was performed with MCScanX to identify different duplication modes, and data were displayed using Circos (Krzywinski et al., 2009; Wang et al., 2012; Qiao et al., 2019). Additionally, TBtools was used to compute the Ka (nonsynonymous) and Ks (synonymous) substitution ratios for *TLP* gene pairs (Chen et al., 2020).

### 2.3 Multi-tissue expression profile analysis of *PbrTLPs*

To analyze *PbrTLPs* expression patterns, transcriptomic data from various pear tissues were collected, encompassing different fruit developmental stages and pollen growth stages (Zhou et al., 2016; Wang et al., 2023). Both transcripts per million (TPM) and reads per kilobase of transcript per million mapped reads (RPKM) were used as measures of transcript levels. TPM or RPKM values (**Supplementary Table S1**) were log_2_-transformed using TBtools, and the maximum expression level for each tissue was normalized to 1 (Chen et al., 2020).

### 2.4 quantitative RT-PCR

Pear (*Pyrus bretschneideri* ‘Dangshansuli’) leaves were collected from the Baima orchard of Nanjing Agricultural University. Total RNA was isolated and reverse transcribed utilizing the corresponding kit (Vazyme, China). Primer Premier 5.0 was employed to design qRT-PCR primers for *PbrTLPs* (**Supplementary Table S2**), and primer specificity was verified against the pear genome database (http://peargenome.njau.edu.cn). Because *PbrTLP34* and *35* share 100% CDS identity (**Supplementary Table S3**), a single qRT-PCR primer pair was used for both. qRT-PCR was performed on a LightCycler 480 II (Roche, Germany) with reaction systems described previously (Zhang et al., 2025). PbrUBQ was used as the internal control, and data were calculated using the 2^−ΔΔCt^ method.

### 2.5 Subcellular localization

The CDS of *PbrTLP28* was amplified from pear leaf cDNA via high-fidelity Phusion DNA polymerase (Vazyme, China), and then cloned into pCAMBIA1300-35S: GFP by homologous recombination. Transient expression of the PbrTLP28-GFP fusion in *N. benthamiana* leaves followed the method previously described (Zhang et al., 2024a). Leaf samples were viewed under an LSM900 confocal microscope (Zeiss, Germany). Plasmolysis was induced in *N. benthamiana* leaves using 2 mol/L NaCl solution. FM4-64 and DAPI were used to stain the plasma membrane and nuclei, respectively.

### 2.6 Virus-induced gene silencing assays and black spot disease treatment

Strain activation, artificial inoculation, and VIGS experiments were conducted as described previously (Xing et al., 2023). The top four to five disease-free leaves from the new shoots of ‘Dangshansuli’ were collected, surface-sterilized with 0.2% NaClO for 30 s, and then washed three times with sterile ddH_2_O. The leaves were placed in culture dishes lined with sterilized, moistened gauze. A sterile needle was used to puncture the inoculation sites, and mycelial plugs (approximately 0.5 cm in diameter) were placed mycelium-side down on the wound sites after 1 h. After inoculation, pear leaves were kept at 25 °C in the dark. Samples were collected at multiple time points (24, 48, 72, and 96 h) and frozen at -80 °C for subsequent RNA isolation. Target gene expression was detected by qRT-PCR (**Supplementary Table S2**).

The CDS sequence of *PbrTLP28* was submitted to the VIGS Tool (https://vigs.solgenomics.net/) to identify a specific 300 bp optimal fragment. This fragment was subsequently inserted into the pTRV2 vector (**Supplementary Table S2**). The pTRV2-PbrTLP28, empty pTRV2, and pTRV1 plasmids were transformed individually into *Agrobacterium* GV3101 (Weidi, China). For VIGS, a mixture of pTRV1 and pTRV2-PbrTLP28 (1:1 ratio) was infiltrated into leaves of 35- to 40-day-old ‘Dangshansuli’ seedlings, with an empty-vector mixture (pTRV1 + pTRV2) used as the control. Each treatment group comprised 10 seedlings, and 2–3 leaves per seedling were infiltrated. After infiltration, plants were incubated in darkness for 1 d, transferred to a 25 °C growth chamber for 20 d, and target gene expression was subsequently analyzed (**Supplementary Table S2**). Leaves from control and silenced plants were surface-sterilized and inoculated with fungal plugs for black spot disease. The infected leaves were kept at 25 °C in darkness, mycelial plugs were removed after 2 d, and lesion areas were measured at 6 d post-inoculation.

### 2.7 Statistical analysis

GraphPad Prism 6.0 was used to visualize the data (shown as mean ± SD), and statistical analyses were conducted as follows: Student’s *t*‐test for pairwise comparisons, and one‐way ANOVA with Tukey’s test for multiple group comparisons.

## 3 Results

### 3.1 Characterization and classification of *TLPs* in Rosaceae

Through BLASTP and HMMER screening, combined with conserved thaumatin domain validation using the SMART and InterPro online tools, a total of 177 *TLPs* were characterized from five Rosaceae genomes and systematically named based on their chromosomal distribution (**Supplementary Table S4**). Among them, 40 *TLPs* were identified in pear (*PbrTLPs*), 40 in apple (*MdoTLPs*), 32 in strawberry (*FveTLPs*), 35 in peach (*PpeTLPs*), and 30 in plum (*PmuTLPs*) (**Supplementary Table S4**; **Figure 1A**).

**Figure 1:**
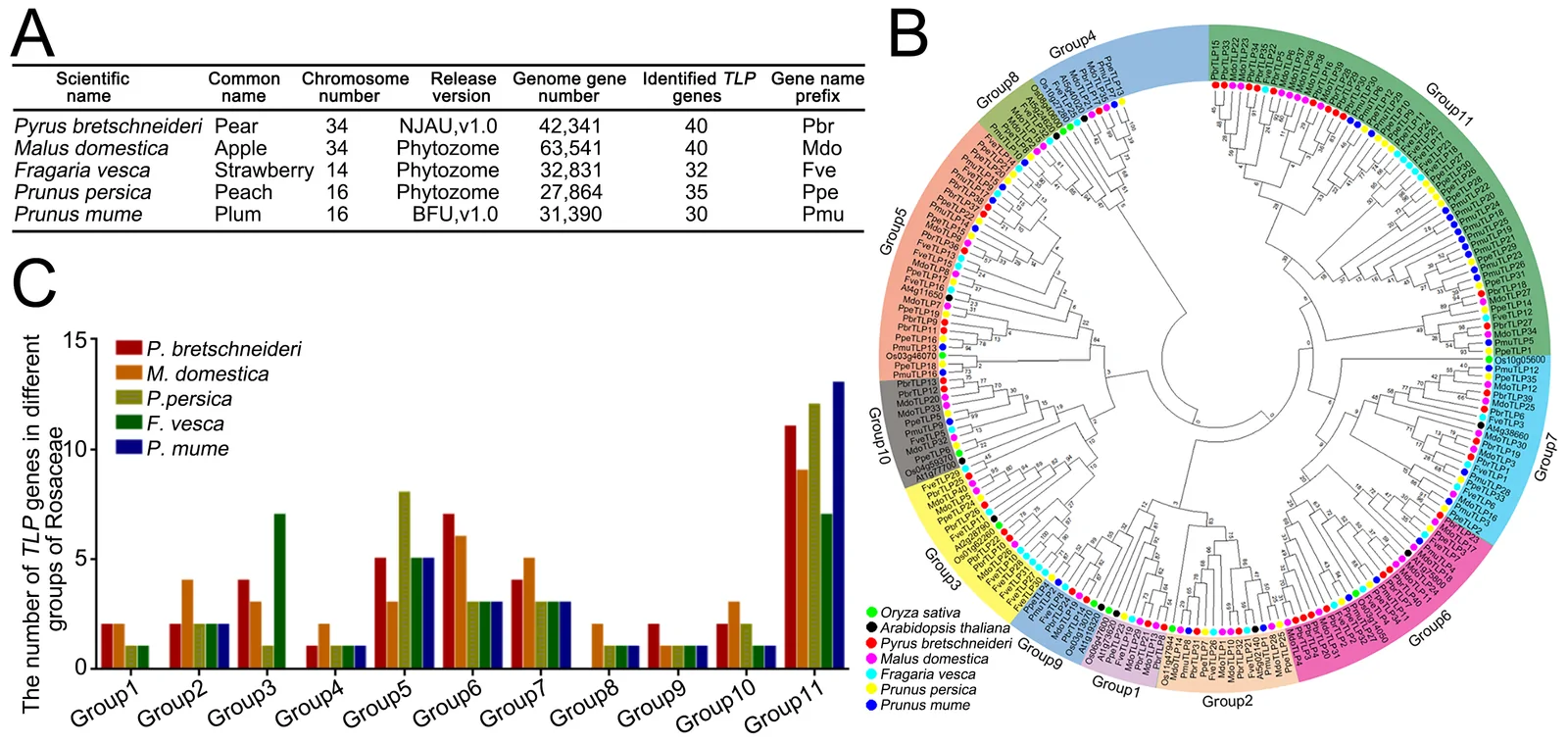
Characterization and phylogenetic analysis of the *TLPs* in Rosaceae. **(A)** Genomic information and *TLP* gene counts of five Rosaceae genomes. **(B)** Phylogenetic relationships of *TLP* families from Rosaceae, rice, and Arabidopsis. Different colored regions indicate the 11 subfamilies of the *TLP* family. Species abbreviations and corresponding dot colors are as follows: At, *Arabidopsis thaliana* (black); Fve, *Fragaria vesca* (cyan); Mdo, *Malus domestica* (pink); Os, *Oryza sativa* (green); Pbr, *Pyrus bretschneideri* (red); Pmu, *Prunus mume* (blue); Ppe, *Prunus persica* (yellow). **(C)** Statistical distribution of Rosaceae *TLP* genes across Groups 1-11.

To explore the evolutionary relationships of Rosaceae TLPs with Arabidopsis and rice TLPs, a phylogenetic tree was constructed using 177 TLP protein sequences from Rosaceae, as well as 10 AtTLP and 10 OsTLP sequences. The phylogenetic analysis classified all TLP proteins into 11 distinct clades, designated Group 1 to Group 11 (**Figure 1B**). The member distribution of each group was as follows: 6 members in Group 1, 12 in Group 2, 15 in Group 3, 6 in Group 4, 26 in Group 5, 22 in Group 6, 18 in Group 7, 5 in Group 8, 6 in Group 9, 9 in Group 10, and 52 in Group 11 (**Figure 1C**). Notably, Group 11 represented a novel, independent clade that was distinct from all TLP subgroups of Arabidopsis and rice (**Figures 1B, C**).

### 3.2 Physicochemical properties analysis of *TLPs* in Rosaceae

Systematic analysis of the physicochemical characteristics of all TLP members (**Supplementary Table S5**) revealed that their lengths ranged between 75 and 914 amino acids (aa), their molecular weights extended from 8,402.76 Da (MdoTLP7) to 99,602.67 Da (PmuTLP8), and their isoelectric points varied from 4.13 (MdoTLP6) to 9.26 (PpeTLP19). The total number of atoms, calculated from the molecular formulas of these Rosaceae TLP proteins, ranged from 1,143 to 13,958. Negative GRAVY scores indicate hydrophilicity, while positive scores indicate hydrophobicity. Across the Rosaceae species, 63.28% of TLP proteins had negative GRAVY scores, indicating that most TLP proteins are hydrophilic. Furthermore, 50.28% of TLP proteins had instability indices below 40, suggesting that these proteins are likely stable. In addition, 92.66% of TLP proteins had aliphatic indices below 80, indicating that most TLP proteins have poor thermostability (**Supplementary Table S5**).

### 3.3 Exon-intron and conserved motif analysis of *TLPs* in Rosaceae

To explore the conservation and divergence of *TLPs* across Rosaceae, we analyzed their genomic sequences and protein characteristics. Exon-intron organization of Rosaceae *TLPs* was examined using the GSDS 2.0. Among them, 38 *TLPs* contained one exon, 79 contained two, 45 contained three, and the remaining 15 had at least four exons (**Figure 2A**; **Supplementary Table S4**). Furthermore, *TLPs* belonging to the same subgroup tended to display similar gene structures. For example, all *TLP* family members in Group 1 of Rosaceae had two exons, whereas those in Group 6 had two to four exons. In addition, conserved motif analysis of TLPs was performed using MEME (**Figure 2B**; **Supplementary Table S6**), revealing that TLP proteins contained five conserved motifs. Among these, Motif 1 was present in 161 TLP proteins, Motif 2 in 166, Motif 3 in 162, Motif 4 in 139, and Motif 5 in 158 TLP proteins. Analysis of the conserved motifs indicated that these five motifs together formed the conserved thaumatin domain.

**Figure 2:**
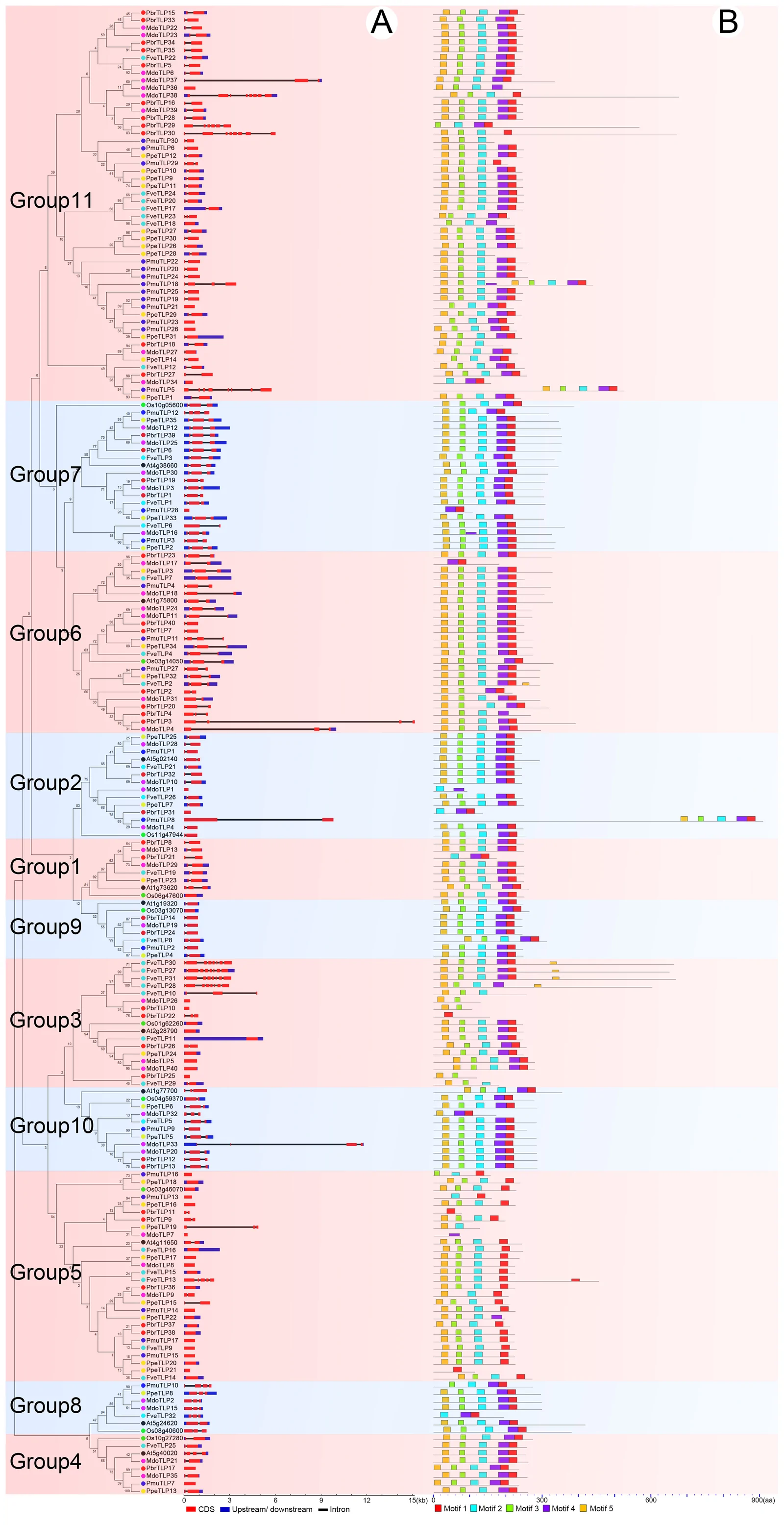
Exon-intron and conserved motif analysis of the *TLP* family in Arabidopsis, rice, and Rosaceae. **(A)** Exon-intron structure analysis of *TLPs*. **(B)** Identification and localization of five conserved motifs in TLP proteins. Background regions of different colors represent the 11 distinct subfamilies.

### 3.4 Chromosomal mapping and evolutionary analysis of *TLPs* in Rosaceae

Gene family expansion can be driven by different duplication patterns (Qiao et al., 2019). We found that the *TLP* family expanded through five duplication modes, with the following proportions: 30.5% whole-genome duplication (WGD), 25.4% tandem duplication (TD), 19.8% dispersed duplication (DSD), 16.9% proximal duplication (PD), and 7.3% transposed duplication (TRD) (**Supplementary Table S7**). Notably, WGD was detected in the five Rosaceae genomes. Among them, 57.5%, 35.0%, 20.0%, 16.7%, and 15.6% of *TLPs* were replicated and retained from WGD in apple, pear, peach, plum, and strawberry, respectively (**Supplementary Table S7**). Additionally, the percentages of TD in pear (25.0%), apple (12.5%), peach (25.7%), plum (30.0%), and strawberry (37.5%) were assessed (**Supplementary Table S7**). The percentages of DSD in apple, pear, peach, strawberry, and plum were 7.5%, 27.5%, 14.3%, 25.0%, and 26.7%, respectively (**Supplementary Table S7**). These findings suggest that WGD significantly contributed to the *TLP* family expansion in apple and pear, whereas TD played an essential role in the expansion within peach, plum, and strawberry.

Through chromosomal mapping and collinearity analysis of *TLP* family members in Rosaceae, we found that 31 *PbrTLPs* were unevenly distributed among 12 chromosomes in pear, with nine genes distributed on scaffolds. Among them, five *PbrTLPs* were located on chromosome 15, and we found 28 homologous gene pairs. In apple, 39 *MdoTLPs* were mapped to 14 of the 17 chromosomes, with five *MdoTLPs* each located on chromosomes 5, 8, and 17, and a total of 45 homologous gene pairs were characterized. For peach, 35 *PpeTLPs* were unevenly distributed across eight chromosomes, with nine *PpeTLPs* located on chromosome 5, and we found 34 homologous gene pairs. In plum, 28 *PmuTLPs* were unevenly distributed across seven chromosomes, with 11 *PmuTLPs* located on chromosome 8, and 29 homologous gene pairs were detected. In strawberry, 32 *FveTLPs* were unevenly distributed across seven chromosomes, with seven *FveTLPs* each located on chromosomes 5 and 7, and a total of 29 homologous gene pairs were identified (**Figure 3A**).

**Figure 3:**
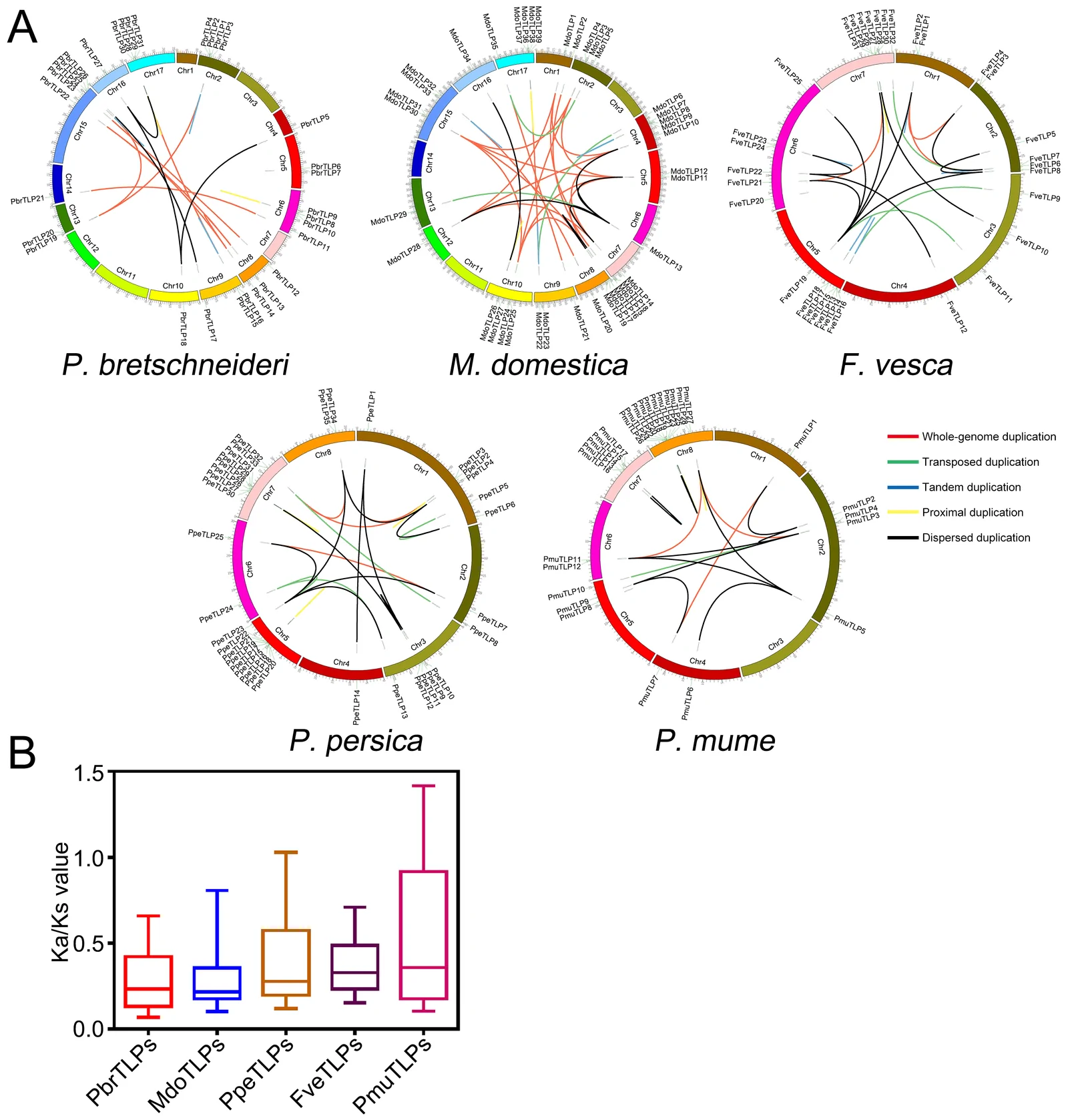
Evolutionary analysis of the *TLP* family in five Rosaceae fruit. **(A)** Chromosomal distribution and duplication mode analysis of *TLPs*. **(B)** Ka/Ks ratio analysis of *TLP* gene pairs in Rosaceae. The X-axis indicates different Rosaceae fruit trees, including pear (*PbrTLPs*), apple (*MdoTLPs*), peach (*PpeTLPs*), plum (*PmuTLPs*), and strawberry (*FveTLPs*), whereas the Y-axis denotes the Ka/Ks value.

To reveal the selective pressures that shaped the evolution of the *TLP* family, we used TBtools to compute the Ka/Ks ratios for every *TLP* gene pair in Rosaceae. The calculated values indicate that, with the exception of five gene pairs (*PpeTLP30*-*PpeTLP31*, *PmuTLP29*-*PmuTLP30*, *PmuTLP24*-*PmuTLP25*, *PmuTLP19*-*PmuTLP20*, and *PmuTLP20*-*PmuTLP22*), all Ka/Ks values were below 1 (**Figure 3B**), indicating that the *TLP* family was primarily shaped by purifying selection during evolution.

### 3.5 Expression analysis of *PbrTLP* family members

To investigate the expression of *PbrTLPs*, we analyzed transcriptomic data from various pear tissues and found that *PbrTLPs* exhibited distinct tissue-specific expression, suggesting different biological functions during pear development (**Figures 4A–D**). Specifically, *PbrTLP7*, *10*, and *19* were highly expressed in seeds; *PbrTLP8*, *23*, and *28* showed elevated expression in leaves; *PbrTLP8* was strongly expressed in roots and stems; *PbrTLP1* and *8* were preferentially expressed in the flesh; and *PbrTLP36* and *32* exhibited relatively higher expression levels during pollen development. Notably, *PbrTLP18* displayed specific and high expression in styles. Additionally, qRT-PCR was conducted to further validate the expression of *PbrTLPs* in pear leaves. The results confirmed that *PbrTLP8*, *23*, and *28* were highly expressed in leaves, with *PbrTLP28* exhibiting the highest expression level (**Figure 4E**), largely consistent with the transcriptomic data. Furthermore, we investigated whether *PbrTLP28* responds to black spot disease in pear leaves. The results demonstrated that *PbrTLP28* was induced in response to black spot disease infection at 24 h post-inoculation, with its expression peaking at 72 h (**Figure 4F**). We also found that *PbrTLP8* and *PbrTLP23* were responsive to black spot disease infection (**Supplementary Figure S1**), indicating that multiple *PbrTLPs* are cooperatively involved in this disease resistance process. Collectively, these results suggest that PbrTLP28 might serve an important function in the disease resistance response of pear leaves.

**Figure 4:**
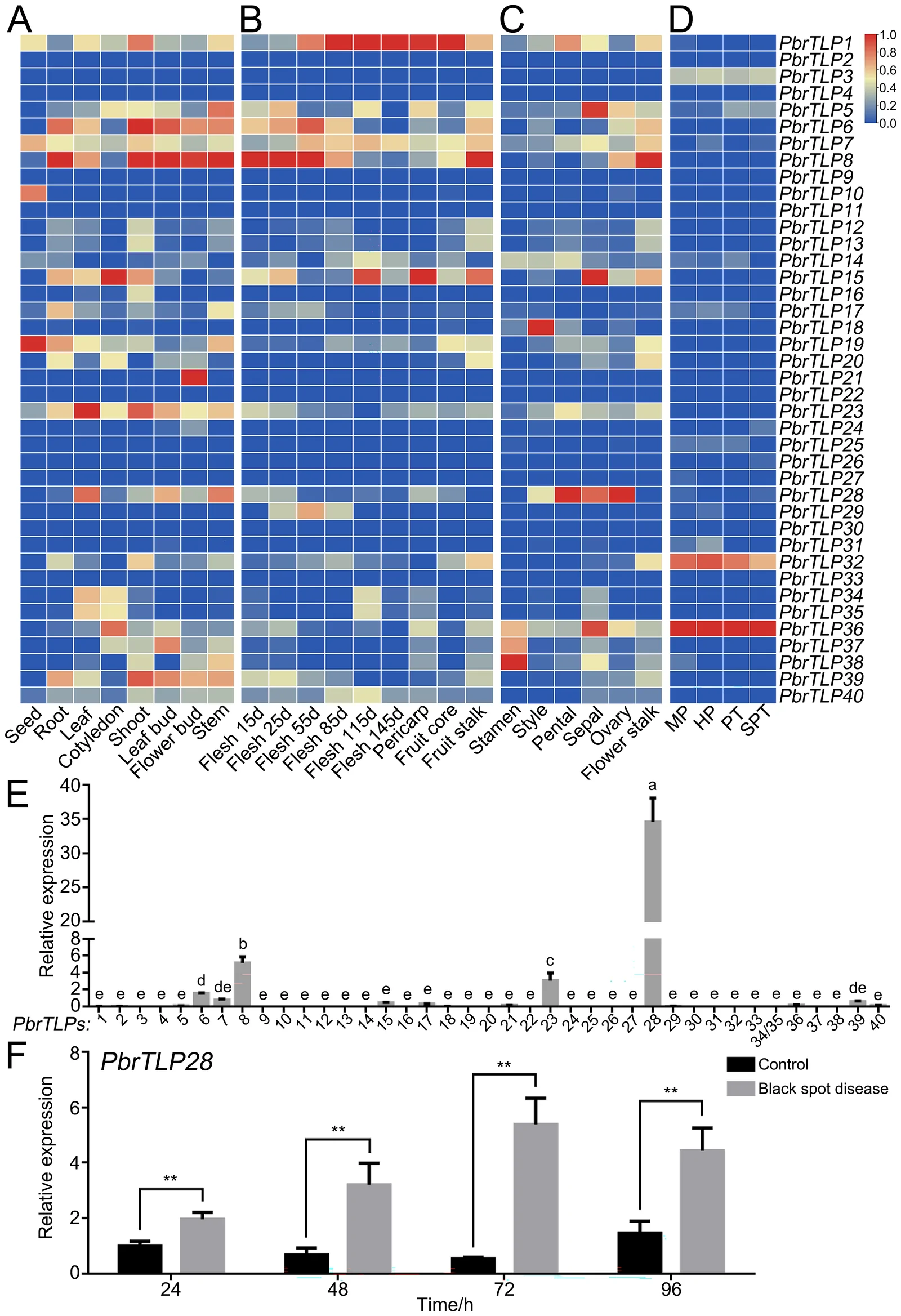
Expression profiles of *PbrTLP* genes. **(A)** Expression heatmap of *PbrTLPs* in different pear tissues. **(B)** Expression heatmap of *PbrTLPs* in floral organs. **(C)** Expression heatmap of *PbrTLPs* during fruit development. MP, HP, PT, and SPT represent mature pollen grains, hydrated pollen, pollen tubes growing 6 h after hydration, and pollen tubes that have stopped growing, respectively. **(D)** Expression heatmap of PbrTLPs during pear pollen growth. MP, HP, PT, and SPT represent mature pollen grains, hydrated pollen, pollen tubes growing 6 h after hydration, and pollen tubes that have stopped growing, respectively. **(E)** Relative expression of *PbrTLPs* in leaves was detected by qRT-PCR. Different letters indicate significant differences among groups (*p* < 0.05, Tukey’s test). **(F)** Black spot disease causes enhanced expression of *PbrTLP28* in pear leaves (“**” indicates *p* < 0.01 by Student’s *t* test).

### 3.6 Subcellular localization of PbrTLP28 proteins

Signal peptide prediction revealed that the N-terminus of PbrTLP28 contains a distinct signal peptide region (**Figure 5A**). Domain analysis further showed that the PbrTLP28 protein has a signal peptide at its N‐terminus and a thaumatin domain at its C‐terminus (**Figure 5B**). To determine its subcellular distribution, we transiently expressed the PbrTLP28‐GFP fusion protein in *N. benthamiana* epidermal cells. PbrTLP28-GFP fluorescence was detected near the plasma membrane and around the nucleus (**Figure 5C**). To confirm that this protein is indeed extracellular, we treated tobacco leaves with 2 M NaCl to trigger plasmolysis. After plasmolysis, PbrTLP28-GFP fluorescence signals were detected between the cell wall and the plasma membrane (**Figure 5D**), consistent with the subcellular localization predictions (**Supplementary Table S4**). Taken together, the above findings suggest that PbrTLP28 is a secreted protein that is synthesized in the endoplasmic reticulum and subsequently transported through the cytoplasm to the extracellular region.

**Figure 5:**
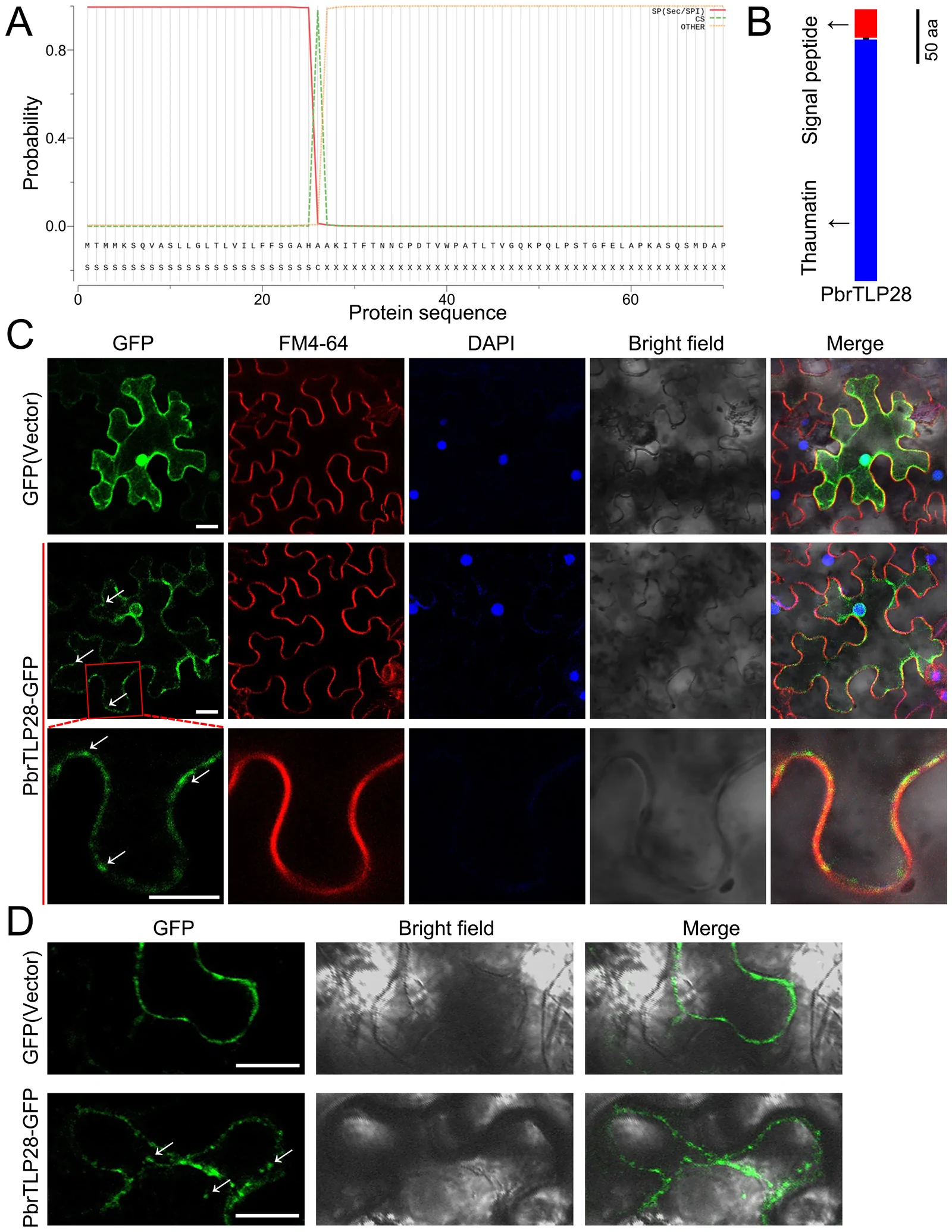
Signal peptide prediction, conserved domain, and subcellular localization analysis of PbrTLP28. **(A)** Diagram showing the signal peptide region within the PbrTLP28 protein sequence. **(B)** Diagram illustrating the conserved domain structure of the PbrTLP28 protein. **(C)** Transient expression of PbrTLP28-GFP in tobacco epidermal cells. FM4–64 and DAPI were used as markers for the cell membrane and the nucleus, respectively. Scale bar = 20 μm. **(D)** Plasmolysis was performed on tobacco epidermal cells. The PbrTLP28-GFP signal is indicated by white arrows. Scale bar = 20 μm.

### 3.7 Silencing the *PbrTLP28* gene increases pear susceptibility to black spot disease

To verify the functional role of PbrTLP28 in resistance to black spot disease, we employed VIGS to suppress its expression. Transient *Agrobacterium*-mediated infiltration of pear leaves with pTRV-PbrTLP28 resulted in efficient silencing of *PbrTLP28*, with transcript levels reduced by 77%, 75%, and 68% in three independent lines (pTRV-1, pTRV-2, and pTRV-3, respectively) relative to the control plants (**Figure 6A**). At 6 days post-inoculation with the black spot pathogen, the pTRV-PbrTLP28 lines exhibited more severe disease symptoms and significantly larger lesion areas than the control plants, indicating enhanced susceptibility to this disease (**Figures 6B, C**). Collectively, these results demonstrate that silencing of *PbrTLP28* attenuates resistance to black spot disease in pear leaves, suggesting that PbrTLP28 functions as a positive regulator of resistance to black spot disease.

**Figure 6:**
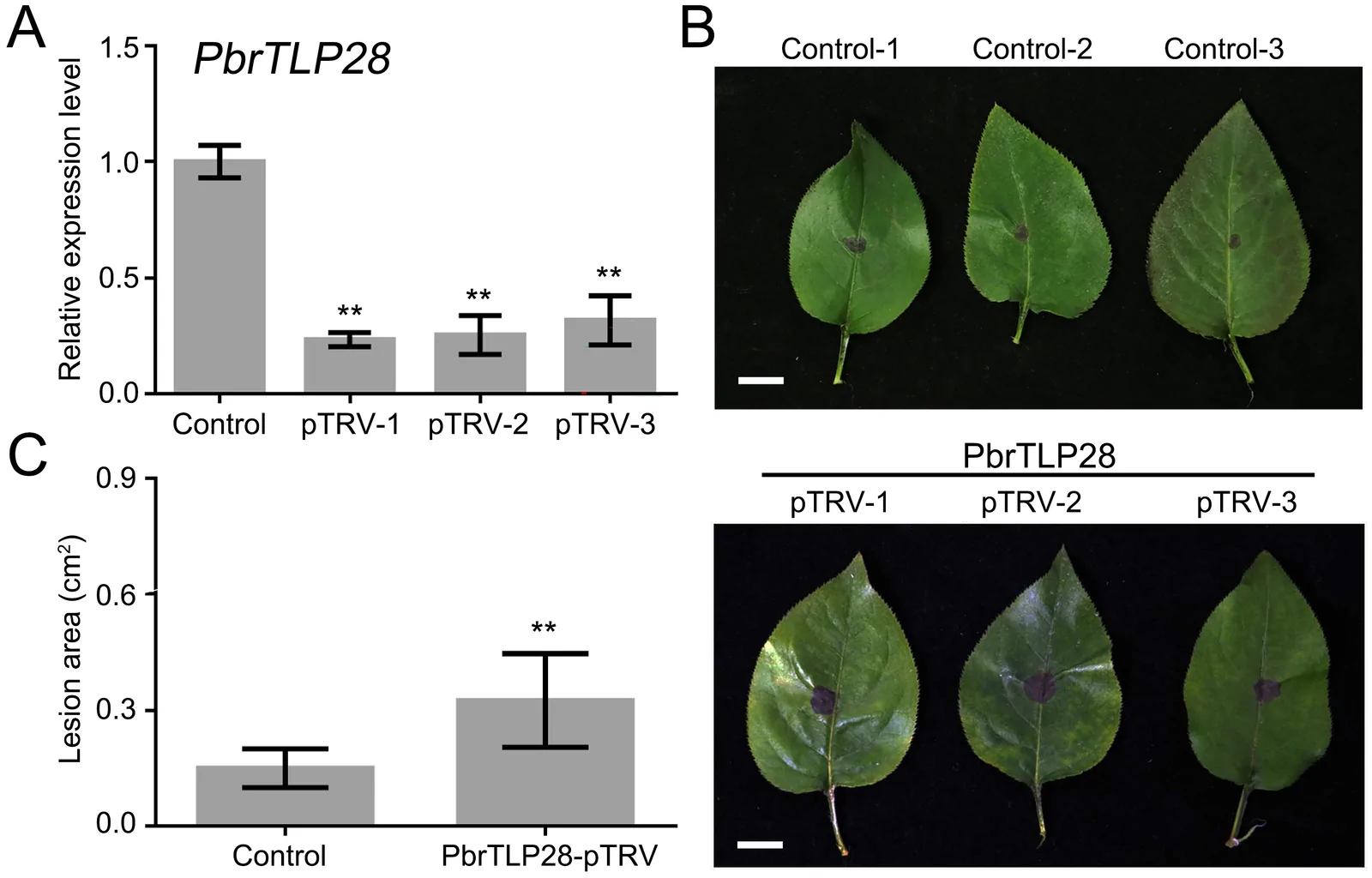
Silencing of the *PbrTLP28* gene increases the susceptibility of pear to black spot disease. **(A)** Relative expression levels of *PbrTLP28* in pear leaves from pTRV-PbrTLP28 lines and control plants. **(B)** Phenotypes of pear leaves from pTRV-PbrTLP28 lines and control plants at 6 days post-inoculation with black spot disease. **(C)** Quantification of lesion areas on pear leaves from pTRV-PbrTLP28 lines and control plants post-inoculation (“**” indicates *p* < 0.01 for Student’s *t* test).

## 4 Discussion

TLPs are known to be induced by a variety of biotic or abiotic factors (Misra et al., 2016; Liu et al., 2020; Zhang et al., 2021) and are involved in plant growth, fruit development, and stress responses (Yu and Griffith, 1999; Kim et al., 2003; O'Leary et al., 2007; Seo et al., 2008; Munis et al., 2010; Zhang et al., 2018b, 2024). While numerous *TLPs* have been identified and functionally characterized in multiple species, the Rosaceae family has received relatively little attention. In this work, we characterized 177 *TLPs* across five Rosaceae species and analyzed the role of PbrTLP28 in pear leaf disease resistance.

Genome sequences are now available for many Rosaceae species (Velasco et al., 2010; Zhang et al., 2012; Verde et al., 2013; Wu et al., 2013; Li et al., 2019), which has greatly facilitated the genome‐wide analysis of *TLP* genes. Here, we identified 177 *TLPs* across five Rosaceae genomes, with pear (40) and apple (40) containing significantly more *TLPs* than strawberry (32), peach (35), and plum (30) (**Supplementary Table S4**; **Figure 1**). Whole-genome analysis indicated that while pear and apple underwent both ancient and recent WGD events, strawberry, peach, and plum lack recent WGD events (Velasco et al., 2010; Wu et al., 2013). Thus, this recent WGD is mainly responsible for the differing *TLP* counts in Rosaceae. Furthermore, all Rosaceae *TLPs* were classified into 11 subfamilies. Compared with rice and Arabidopsis, a novel subcluster (Group 11) was characterized in Rosaceae genomes. Analyses of conserved motifs and gene structures provided additional support for the classification of Rosaceae *TLP* genes (**Figure 2**). Some Rosaceae *TLPs* exhibited loss or gain of exons, introns, and motifs, which may have generated structural diversity in *TLPs* during long-term evolution and consequently led to partial functional divergence (Zhang et al., 2024a; Liu et al., 2025).

Different duplication modes are known to facilitate gene family expansion (Qiao et al., 2019). Duplication mode analysis revealed that WGD contributed largely to the expansion of the *TLP* family in apple and pear, whereas TD served as the major contributor for strawberry, peach, and plum. Overall, the expansion of the *TLP* family in Rosaceae involved multiple duplication modes, among which WGD and TD contributed up to 55.9 % of the expansion, serving as the major driving forces (**Supplementary Table S7**). Moreover, Ks analysis showed that most Ks values in apple and pear were distributed between recent (Ks ~ 0.15-0.3) and ancient (Ks ~ 1.5-1.8) WGD, whereas those in strawberry, peach, and plum were predominantly clustered within the ancient WGD (**Supplementary Table S8**). These results demonstrate that recent WGD markedly promoted the expansion of the *TLP* family in apple and pear, further confirming that recent WGD is the primary cause of the greater number of *TLPs* in apple and pear relative to strawberry, peach, and plum, a phenomenon also reported for other gene families (Liu et al., 2024, 2025). Furthermore, most Ka/Ks ratios calculated here were below 1 (**Figure 3B**; **Supplementary Table S8**), implying that purifying selection is the primary driver of evolution in the *TLP* family. This pattern may be associated with the long-term domestication and breeding of high-quality disease-resistant pear cultivars.

The expression of *TLPs* is modulated by multiple factors, such as pathogen attack, osmotic stress, and phytohormones (Misra et al., 2016; Li et al., 2020; Liu et al., 2020; Zhang et al., 2021; Li et al., 2022; Sharma et al., 2022). In addition, some *TLPs* exhibit distinct tissue specificity or are highly expressed at specific developmental stages (Zhang et al., 2024b). For example, *AtTLP1* shows root vascular‐specific expression upon colonization by nonpathogenic *Pseudomonas* (Léon-Kloosterziel et al., 2005). Notably, the PsTL1 protein from *Pyrus serotina* specifically accumulates during style development (Sassa and Hirano, 1998), and heterologous transformation of the *PsTL1* promoter into tobacco revealed GUS activity in the pistil, anther, and pollen (Sassa et al., 2002). Furthermore, many TLP proteins are highly expressed during fruit ripening in certain plants, such as apple, banana, and cherry (Fils-Lycaon et al., 1996; Kim et al., 2003; Ho et al., 2007). These results demonstrate that *TLP* expression is closely correlated with various plant growth and developmental processes. In this study, we found that *PbrTLP18* was specifically and highly expressed in pear styles, whereas *PbrTLP36* and *PbrTLP32* were strongly expressed during pollen development (**Figures 4A–D**). Notably, *PbrTLP28* was highly expressed in pear leaves and was rapidly upregulated following black spot disease infection (**Figures 4E, F**). Collectively, these findings indicate that PbrTLP28 is essential for conferring resistance to diseases in pear leaves.

Multiple studies have demonstrated that TLPs participate in plant defense responses and enhance resistance to various fungal pathogens (Ojola et al., 2018; Li et al., 2020; Sun et al., 2020; Zhang et al., 2021). For example, grape *VqTLP29* is induced by *E. ampelina*, *Erysiphe necator*, and *B. cinerea*, and overexpressing *VqTLP29* in Arabidopsis conferred resistance to *E. necator* (Yan et al., 2017). Garlic *AsPR5* exhibited high expression under *Fusarium oxysporum* infection, and its heterologous overexpression in Arabidopsis conferred improved resistance to *B. cinerea* (Rout et al., 2016). Cotton *GhTLP19* was significantly upregulated upon *V. dahliae* infection, and silencing of *GhTLP19* via VIGS reduced resistance to *V. dahliae* (Li et al., 2020). In this study, silencing of *PbrTLP28* resulted in significantly larger lesion areas on pear leaves inoculated with the black spot pathogen, and this phenotype was consistently validated across different lines (**Figure 6**). Therefore, silencing *PbrTLP28* attenuated pear resistance to black spot disease, indicating that this gene functions in disease resistance. Moreover, the majority of Rosaceae TLP proteins contain a signal peptide, suggesting their likely secretion into the extracellular space (**Supplementary Table S4**). Concurrently, subcellular localization experiments further demonstrated that PbrTLP28 is a secreted protein present in the extracellular space (**Figure 5**). Similarly, previous findings also demonstrated that TLP proteins in wheat, peanut, and tea are localized to the extracellular space (Wang et al., 2010; Singh et al., 2013; Zhang et al., 2018a; Muoki et al., 2021; Sharma et al., 2022). Collectively, these results indicate that *PbrTLP28* expression is markedly upregulated upon black spot disease infection in pear leaves, and the secreted PbrTLP28 protein enhances pear resistance to this pathogen.

## Data Availability

The original contributions presented in the study are included in the article/**Supplementary Material**. Further inquiries can be directed to the corresponding authors.

## Supplementary material

Supplementary Table 1 Summary of transcriptomic sequencing data from different pear tissues.

Supplementary Table 2 List of primers used in this work.

Supplementary Table 3 Comparative analysis of *TLP* coding sequence identity in pear.

Supplementary Table 4 Basic information of *TLPs* in five Rosaceae species.

Supplementary Table 5 Physicochemical analysis of TLP proteins in five Rosaceae species.

Supplementary Table 6 Analysis of conserved motif logos of TLP proteins identified by MEME.

Supplementary Table 7 Numbers of *TLPs* derived from different duplication origins in five Rosaceae species.

Supplementary Table 8 Ka, Ks and Ka/Ks analysis of *TLP* duplicated genes from five Rosaceae species.

Supplementary Figure 1 Black spot disease causes enhanced expression of *PbrTLP8 and PbrTLP23* in pear leaves.
